# Spatial Interactions between Successive Eye and Arm Movements: Signal Type Matters

**DOI:** 10.1371/journal.pone.0058850

**Published:** 2013-03-19

**Authors:** Christopher D. Cowper-Smith, Jonathan Harris, Gail A. Eskes, David A. Westwood

**Affiliations:** 1 Department of Psychology and Neuroscience, Dalhousie University, Halifax, Nova Scotia, Canada; 2 School of Health and Human Performance, Dalhousie University, Halifax, Nova Scotia, Canada; 3 Department of Psychiatry, Dalhousie University, Halifax, Nova Scotia, Canada; University of Muenster, Germany

## Abstract

Spatial interactions between consecutive movements are often attributed to inhibition of return (IOR), a phenomenon in which responses to previously signalled locations are slower than responses to unsignalled locations. In two experiments using peripheral target signals offset by 0°, 90°, or 180°, we show that consecutive saccadic (Experiment 1) and reaching (Experiment 3) responses exhibit a monotonic pattern of reaction times consistent with the currently established spatial distribution of IOR. In contrast, in two experiments with central target signals (i.e., arrowheads pointing at target locations), we find a non-monotonic pattern of reaction times for saccades (Experiment 2) and reaching movements (Experiment 4). The difference in the patterns of results observed demonstrates different behavioral effects that depend on signal type. The pattern of results observed for central stimuli are consistent with a model in which neural adaptation is occurring within motor networks encoding movement direction in a distributed manner.

## Introduction

In everyday tasks such as reading, driving, or eating, we engage in *sequences* of spatially directed movements. Whereas each movement might have an independent goal, the planning and execution of *consecutive* movements made with the same effector is likely to rely on overlapping spatial representations. As such, it is of interest to explore the spatial interactions that occur between movements made close together in time.

Such interactions have been explored extensively for orienting responses, in which participants respond to a *target* stimulus after a preceding *cue* stimulus. As initially described by Posner and Cohen [1], for cue-target onset asynchronies of approximately 300 ms (or more), responses are slower to targets appearing at cued versus uncued locations – a phenomenon later given the name ‘inhibition of return,’ (IOR) to reflect the selective bias against responding to previously signalled locations [2].

Many different task parameters have been adopted in order to study IOR, however two commonly manipulated variables are: (1) response type (e.g. manual or saccadic) and (2) signal type (e.g. peripheral onset or central arrowhead) [1]–[7]. Although the presence or absence of IOR has been studied across all possible combinations of these response and signal types [3], the spatial distribution of IOR, i.e. the pattern of RTs observed for targets that are presented at, intermediate to, or opposite the cued location, remains incompletely characterized for different signal types. In particular, although the spatial distribution of IOR is well established when peripheral stimuli are used (as described below) [8]–[16], the same cannot be said when central arrowhead stimuli are used. This is because previous studies using central signals have relied on the use of two target locations, typically aligned to the left and right of fixation (e.g. [3], [4], [17]). While these conditions enable one to determine the presence and magnitude of an RT difference between the cued and uncued target locations, they do not enable a comprehensive analysis of the spatial properties of the putative IOR phenomenon. Therefore, previous work that has inferred the presence of IOR while using central signals and only two possible target locations, may have missed important data points regarding the spatial properties of the (putatively observed) IOR phenomenon. In particular, it remains unclear whether IOR experiments using central signals will reveal similarly distributed behavioural effects to that observed with peripheral signals.

Some lines of evidence provide reason to believe that different forms of IOR can be observed depending on the type of signal used to prompt responses. For example, some scholars have argued that peripheral and central stimuli can be used to reveal the sensory/attentional and motor forms of IOR respectively (e.g. [3]–[7], [17]–[19]). Specifically, when consecutive *peripheral* stimuli are used, IOR might inhibit either (1) information received from a particular location in space (i.e., sensory/attentional processing) or (2) the production of any required response (i.e., motor processing). In contrast, when *central* stimuli are used, any inhibition attached to a peripheral location cannot disrupt the processing of the imperative stimulus, but response-based inhibition is possible. Correspondingly, IOR observed with central stimuli might be considered to affect motor-based processes [3]–[7], [17]–[19]. Notably however, other scholars have shown that late-stage attentional processes can be tied to the generation of motor responses (e.g. a movement of attention that immediately precedes the execution of an eye or arm movement) [20]–[21]; it is therefore possible that motor, late-stage attentional, or some combination of these processes are affected by IOR when central signals are used to prompt motor responses. (Notably however, if late-stage attentional processes that are tied to the execution of a movement are inhibited when central stimuli are used, they are nonetheless likely to be different from the sensory/attentional processes that are inhibited when peripheral stimuli are used. For example, when simple detection responses are required, IOR is only observed when peripheral, but not central target stimuli are used; this observation indicates that the presentation of the arrow alone is insufficient to reveal IOR, and moreover, that peripheral target stimuli can be used to reveal a spatially localized deficit in sensory/attentional processing).

If different sensory/attentional and response-based forms of IOR can in fact be dissociated by signal type (at least in part), then given that the sensory/attentional and motor response systems represent space differently (depending on the stage of processing affected) (cf., [1], [7], [22]–[24]), it is possible that different spatial distributions of IOR will be observed depending on signal type. The spatial distribution of IOR observed with peripheral stimuli is associated with a clear monotonic relationship between response latency and the angular spatial offset between the first and second stimuli [8]–[16]: the latency of a response to the second stimulus is greatest when it shares the same location as the first stimulus (i.e., a 0° offset), and decreases as the spatial offset between stimuli increases to 180°. This monotonic pattern of IOR has been established by examining RTs across many different angular offsets ranging between 0° and 180°, although many studies have adopted 0°, 90° and 180° offsets. As discussed earlier, the spatial distribution of RTs observed when central signals are used remains un-established. On one hand, if IOR is similar when peripheral and central stimuli are used (as suggested by previous research using only two target locations) (e.g., [3]) then one would expect to observe similar spatial distributions of RTs independent of signal type. On the other hand, if sensory/attentional and motor forms of IOR can be dissociated, at least in part by signal type, then it is possible that different spatial distributions will be observed when motor responses are instructed by central versus peripheral stimuli.

Using variations of the traditional center-out consecutive target paradigm (where participants respond to the first signal and then return to center before responding to the second signal [3]), we examined the pattern of RTs observed in four experiments when participants were required to make consecutive eye (E1 and E2) or arm (E3 and E4) movements to either peripheral (E1 and E3) or central (E2 and E4) stimuli (because consecutive responses are required, this task is referred to as a target-target paradigm (e.g., [3])). We predicted that if signal type plays an important role in shaping the pattern of RTs observed, then the pattern of RTs observed should vary as a function of signal type, but be relatively independent of the effector system used to respond. For the experiments involving peripheral stimuli, based on prior work [8]–[16], we predicted a monotonic spatial relationship between RT and target-target spatial offset (0° >90° >/ = 180°; see the introduction to E1 for more details) for saccades (E1) and reaching movements (E3) alike. If the spatial topography of IOR (as traditionally defined) is insensitive to signal type, then one would expect to observe a similar monotonic spatial distribution of RTs across all offset conditions when central arrowhead signals (rather than peripheral onsets) were used to prompt responses. To anticipate the results, different spatial patterns of RTs were observed when peripheral and central signals were used to prompt consecutive responses respectively. These results are interpreted and discussed in the context of the possible mechanisms and functions underlying the observed RT patterns.

## Experiment 1: Peripheral Target–Eye

In E1 we aimed to extend the monotonic spatial pattern of IOR (cf. [8]–[16]) observed in previous cue-target studies to a target-target task that required participants to make two consecutive saccades to peripheral target stimuli. Previous cue-target studies revealed a monotonic pattern of RTs (0°>90°>/ = 180°), where (1) RTs are greatest at the cued location, (2) RTs drop off sharply as the cue-target offset increases from 0° to 90°, and (3) RTs do not increase, but will either remain stable or continue to decrease slightly (both patterns fit the monotonic definition) from 90° to 180°. Consistent with previous work examining the spatial distribution of IOR (e.g., [5], [15], [16], [22]) we used four target locations (up, down, left and right of fixation) that allowed us to vary the degree of directional offset between the first and second stimuli from 0° (i.e., same direction) to 180° (opposite direction), in 90° increments. Confirmation of the monotonic IOR pattern in this paradigm was important, in order to subsequently compare the topography of RTs when central rather than peripheral signals were used in E2.

### Materials and Methods

All experimental procedures were approved by the local research ethics board in the Department of Psychology and Neuroscience at Dalhousie University, and participants in all studies provided written informed consent.

#### Participants

Nineteen (11 female, 8 male) undergraduate students participated in E1. All participants were recruited through the Department of Psychology subject pool at Dalhousie University. Participants were right handed, had normal or corrected-to-normal vision and reported no history of visual, motor, or neurological abnormalities.

#### Apparatus and Stimuli

Stimuli were displayed using Experiment Builder v1.3 software (Eyelink II; SR Research, Mississauga, Ontario, Canada). Eye position was monitored with an EyeLink™II (SR Research, Osgoode, ON) eye-tracking system (sampling rate = 500 Hz; spatial precision <0.01; spatial accuracy <0.8 root mean square error). Calibration of the EyeLink II was carried out in the same horizontal viewing plane that was used to display the target stimuli. Participants were seated at a viewing distance of approximately 58 centimetres from the screen.

**Figure 1:**
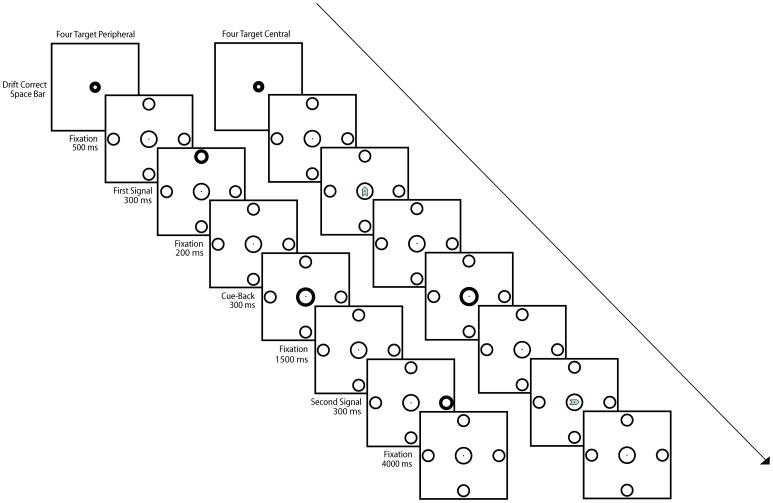
Sequence of Stimuli. Example stimuli and sequence timing from a single trial when peripheral (E1 and E3) and central (E2 and E4) signals were used to prompt responses. Each trial began with a drift correction procedure that required the participant to press the space bar with their left hand while fixating within the fixation circle. The fixation array was then displayed for 500 ms after which the first signal (S1) was displayed for 300 ms. Following the offset of S1, fixation was displayed for 200 ms followed by a cue-back stimulus (change of the fixation circle outline from 4 px to 8 px weight) for 300 ms. The fixation array (with all circles in 4 px weight) was again displayed for 1500 ms, providing ample time for participants to return their eye/arm to center prior to the onset of the second signal (S2). The S2 stimulus was added for 300 ms, followed again by an inter-trial interval of 4 seconds during which the fixation array was displayed. Participants were instructed to maintain fixation throughout the trial. During each trial, participants were asked to make an eye or arm movement to touch the center of the signalled targets as quickly and accurately as possible, and to return to center upon display of the cue-back, as well as after completing their S2 response. The overall fixation array was present throughout each trial, thereby providing a stable stimulus background while S1, S2, and the cueback were overlain as described above.

Stimuli consisted of a fixation circle 3.15° in diameter that was surrounded by 4 equidistant peripheral placeholders (circles that were 2.5° in diameter; Figure 1). Placeholders were spaced 4.6 degrees away from fixation (measured from the center of fixation to the center of the placeholder) and were separated by 90° from each other (i.e., up, right, down, left). The outlines of the central fixation circle and peripheral placeholders were presented with a 4 px weight on a 30-inch ELO touch screen LCD monitor (11.7 ms response time; Elo TouchSystems, Menlo Park, California, USA).

#### Procedure

The Eyelink®II system was calibrated using a 9 point routine. Participants practiced trials selected randomly from the main experiment until the successful completion of 8 consecutive trials. Participants were required to complete these trials without any error feedback, as described below. Trials consisted of two consecutive signals (S1 and S2) where the border of a peripheral placeholder temporarily changed from a 4 px line-weight to an 8 px line-weight. Participants were instructed to saccade to the target with the bolded outline. S1 and S2 indicated each of the four possible target locations with equal probability (0.25), creating a total of 16 equally possible S1/S2 pairings. These pairings therefore signalled consecutive saccadic responses that were offset from each other by 0°, −90°, +90°, or 180. After practice was completed, each S1/S2 pairing was presented 12 times for a total of 192 trials which were divided into two runs of 96 trials separated by a short break. S1/S2 pairings were randomized on a trial-by-trial basis.

The timing of stimuli within a single trial for E1 is shown in Figure 1. Participants were instructed to make a saccade toward and fixate the signalled targets as quickly and accurately as possible, and to return their eyes to center upon display of the cue-back as well as after completing their S2 response. If participants did not respond within 1.5 seconds to S1 or S2, if they did not return their eyes to center between S1 and S2, or if they failed to maintain fixation during the fixation stimulus (immediately prior to S1 or S2), an error message was displayed, the trial was aborted, and was not recycled. Data from aborted trials were excluded from all subsequent analyses. Less than 5% of trials were aborted due to a slow response, a failure to return their eyes to center between S1 and S2, or a failure to maintain fixation. The key dependent measure for all experiments reported herein is the reaction time (RT) for the response to the *second* target in the sequence (i.e., S2 RT).

#### Data analysis

The description of data analyses here applies to all four experiments in the present paper. Trials with S2 RTs less than 100 ms (anticipation) or greater than 1000 ms (miss) were flagged during data processing and excluded from all analyses. Consistent with previous work [3], data were also flagged and excluded if the S1 response was greater than 500 ms. Trials in which participants moved their eyes to the wrong S2 target were flagged as directional errors. Directional error trials were eliminated from the main RT analysis but were tallied and analyzed to determine the possibility of speed-accuracy tradeoffs. The frequency of anticipation, miss, and directional errors out of the total trials (N = 192) accounted for less than 5% of trials.

Because we were interested in demonstrating that the typical monotonic pattern of RTs observed previously in cue-target IOR studies (0°>90°>/ = 180° [8]–[16]) also occurs in a target-target paradigm, mean RTs for S2 saccades were analyzed using a repeated-measures ANOVA (alpha = 0.05) with the sole factor of offset (i.e., the angular offset between S1 and S2∶0°, 90° or 180°). Note that +90° and −90° conditions were collapsed into a single 90° condition for the purpose of these analyses. Mauchly’s test was used to test the assumption of sphericity (alpha = 0.05); if sphericity was violated, the Greenhouse-Geisser correction was applied and adjusted degrees of freedom are reported. All offset conditions were compared using planned pairwise comparisons (alpha = 0.01), in order to determine if a monotonically declining pattern of RTs was observed. Directional errors within each offset condition (0°, 90°, or 180°) were analyzed to detect possible speed-accuracy trade-offs, in order to determine if a reduction in movement accuracy accompanied reduced reaction times. The frequency of directional errors in each offset condition was first used to calculate a percent error rate for each offset condition; a repeated measures ANOVA with the sole factor of offset was then conducted on these values.

### Results

#### Errors

Error rates were calculated independently within each offset condition. Directional errors were minimal and accounted for 0.27% (range = 0–1.6%, SD = 0.4%), 0.78% (range = 0–2.1%, SD = 0.9%), and 0.33% (range = 0–1.5%, SD = 0.5%) of the total trials in the 0°, 90°, and 180° conditions respectively. Because error rates were less than 1% in each offset condition, they were not analyzed further.

#### Saccadic reaction time to S2

**Figure 2:**
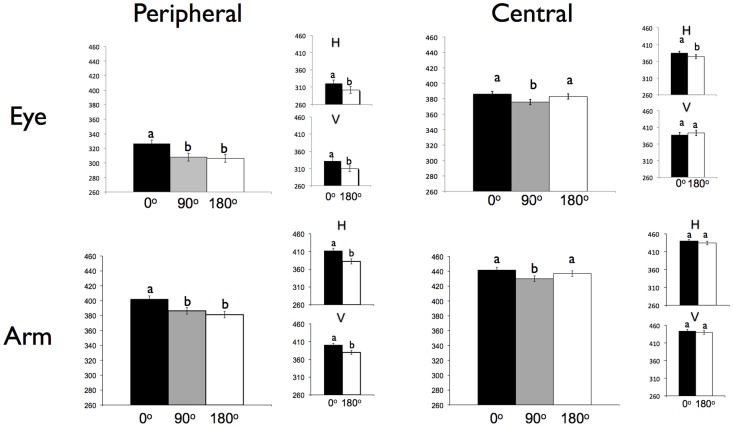
Reaction Times. Mean saccadic reaction times to the second signal (S2) for Experiments 1–4 are presented on the Y axis with RTs for each offset condition (0°, 90°, and 180°) presented as separate bars. Experiment 1 (Peripheral – Eye) is shown in the upper left; Experiment 2 (Central - Eye) is shown in the upper right; Experiment 3 (Peripheral - Arm) is show in the lower left; Experiment 4 (Central - Arm) is shown in the lower right. Conditions labelled with different letters (a, b, or c) are significantly different from each other. Error bars show within-subjects 95% confidence intervals, as described by Masson [41] using the Offset x Subject MSE term. H stands for horizontal axis; V stands for vertical axis.

Saccadic S2 RTs are shown in Figure 2 (“Peripheral - Eye”). A main effect of offset was observed, F(2,18) = 15.56, *p*<0.001. Pairwise comparisons revealed slower RTs in the 0° relative to 90°, F(1,18) = 17.87, *p*<0.001, and the 0° relative to 180°, F(1,18) = 17.88, *p*<0.001 offset conditions. Reaction times were not significantly different for the 90° and 180° offsets, F(1,18) = 0.43, *p* = 0.52.

Many previous IOR studies contained only two possible target locations to the left and right of fixation (e.g. [3], [4], [17]). To compare our results to these studies, we analyzed separately those trials where the S1–S2 responses were restricted to the horizontal (left or right), and vertical (up or down) axis. We further compared amongst each of the possible 90° offset combinations (up/right = upper right [UR], right/down = lower right [LR], down/left = lower left [LL], left/up = upper left [UL]) to determine if the overall faster S2 RTs for the 90° offset condition were attributable to specific target combinations.

Zero degree offset responses were significantly slower than 180° offset responses within both the horizontal, F(1,18) = 7.42, *p*<0.05 and vertical F(1,18) = 12.09, *p*<0.005 axes. No differences in the magnitude of IOR (where magnitude of IOR = RTs for “same” S1 & S2 trials minus RTs for “different” S1 & S2 trials) was observed between any of the possible 90° offset combinations (UL, UR, LR, LL), F(3,18) = 0.55, *p* = 0.65, indicating that the overall faster RTs for the 90° offset condition were not driven by any specific 90° S1/S2 combination(s).

### Discussion

As predicted, the results of E1 extend the monotonic pattern of IOR (0°>90°>/ = 180°) observed in previous cue-target studies using peripheral stimuli to a target-target task. The results from E1 were used as a baseline against which to compare the data from E2 in which central stimuli were adopted.

## Experiment 2: Central Target–Eye

Experiment 2 mirrored E1 except the peripheral signals (S1 and S2) were replaced by central signals. Similar to E1, peripheral placeholders were continuously present in E2, ensuring that participants made responses that were metrically identical to those in E1, i.e. with a similar movement direction and amplitude. If the same spatial distribution of RTs is observed between E1 and E2, then it would be reasonable to conclude that IOR is similarly implemented independent of signal type. If markedly different patterns of RTs are observed between E1 and E2, it would suggest an important role of signal type.

### Method

#### Participants

Twenty (15 female, 5 male) undergraduate students participated in E2. All participants were recruited through the Department of Psychology subject pool at Dalhousie University. All participants were right handed, had normal or corrected-to-normal vision and reported no history of visual, motor, or neurological abnormalities.

#### Apparatus and stimuli

The stimulus configuration and sequence was identical to that used in E1 except rather than using peripheral signals (i.e., bolded placeholders), eye movements were signalled to continuously present peripheral placeholders using arrowheads displayed at fixation (Figure 1). Arrows were 1.5 visual degrees in length and 0.5 degrees in width.

#### Procedure and data analyses

The procedure was identical to E1 except that eye-movement signals in each trial consisted of arrowhead stimuli presented at central fixation rather than peripheral stimuli. The protocol for RT and error data analyses were equivalent between E1 and E2. The frequency of anticipation, miss, and directional errors (all removed from subsequent analyses) accounted for less than 5% of trials.

### Results

#### Errors

Directional errors accounted for 0.76% (range = 0–3.6%, SD = 1.0%), 1.3% (range = 0–5.7%, SD = 1.8%), and 0.89% (range = 0–2.6%, SD = 1.2%) of the total trials in the 0°, 90°, and 180° conditions respectively. No significant difference was observed in the directional error rates between offset conditions, F(2,38) = 2.68, *p* = 0.08.

#### Saccadic reaction time to S2

Saccadic RTs are shown in Figure 2 (“Central - Eye”). A main effect of offset was observed, F(2,19) = 10.6, p<0.001. Pairwise comparisons revealed slower RTs for 0° relative to 90° offset conditions, F(1,19) = 50.1, *p*<0.001 and for 180° relative to 90° offsets, F(1,19) = 8.8, *p*<0.01. Reaction times were not significantly different for 0° and 180° offsets, F(1,19) = 1.2, *p* = 0.28. In order to compare the spatial distribution of IOR observed in E2 to that observed in E1, we conducted a 2×3 mixed ANOVA with factors of signal type (peripheral [E1] or central [E2]) and offset (0°, 90°, and 180°). Significant main effects of signal type F(1,18) = 31.3, p<0.001, and offset, F(2,36) = 17.9, *p*<0.001, were observed. Moreover, a significant interaction between signal type and offset was observed F(2,36) = 7.9, *p*<0.001, indicating a difference in overall spatial topographies between experiments.

The lack of difference between RTs for 0° and 180° offsets is inconsistent with Taylor and Klein’s [3] study which showed significantly slower RTs for 0° versus 180° offsets. However, as discussed earlier, those authors employed a task with only two targets, on the left and right of fixation. Similar to E1, we therefore analyzed the 0° and 180° offset conditions for the horizontal and vertical axes separately. We further compared each of the 90° offset combinations (UL, UR, LR, LL) to determine if the overall faster S2 RTs observed could be accounted for by any particular 90° S1/S2 combination(s).

Consistent with Taylor and Klein [11], within the horizontal axis, RTs were greater in the 0° compared to 180° offset condition, F(1,19) = 6.68. *p = *0.018. Within the vertical axis however, RTs were not significantly different for 0° and 180° conditions, F(1, 19) = 1.4, *p = *0.25. No differences in the magnitude of IOR (magnitude = same S1/S2 location RTs – different S1/S2 location RTs) was observed between any of the possible 90° offset combinations (UL, UR, LR, LL), F(1,19) = 1.7, *p = *0.18, indicating that the overall faster RTs for the 90° offset condition were not driven by a specific combination of first and second saccade directions.

### Discussion

The results of E2 demonstrate a distinct topography of RTs to that observed in E1, and indeed in all previous IOR research [8]–[16]. Unlike the monotonic pattern observed in E1 (0°>90° = 180°), in E2 we observed a non-monotonic pattern of RTs where 90° offsets were faster than both 0° and 180° offsets. This empirical observation is important, because IOR is commonly defined by its spatial topography [8]–[16]; as such, the present result is inconsistent with the currently established spatial definition of IOR.

Interestingly, when we grouped the data from E2 into 0° and 180° offset trials that were aligned with the horizontal and vertical axes, we found a significant difference between 0° and 180° offsets for the horizontal but not vertical axis. In contrast with the majority of previous research that has examined IOR across only two target locations aligned to the left and right of fixation, the results of E2 therefore highlight the importance of analyzing RT effects across multiple spatial locations aligned in different axes.

Note that in the present experiments, we used a stimulus array consisting of targets aligned with the cardinal axes around a central fixation point, as is typical of many other studies. One consideration arising from this stimulus array relates to the possibility that targets on the horizontal meridian might be represented on opposite sides of the nervous system, whereas targets aligned with the vertical meridian might be represented bilaterally. Following this logic, 90° offset conditions would always involve a transition in control from a unilateral to bilateral representation, or vice versa. It is conceivable that this transition might account for the decreased latency of movements in the 90° offset conditions as compared to 0° and 180° conditions. However, this explanation is unlikely for several reasons. First and foremost, this line of reasoning would apply equally well to the tasks in E1 and E2, but these experiments yield different results with respect to the comparison of 0°, 90°, and 180° offset conditions. Nevertheless, in order to rule out concerns about the use of targets aligned with the horizontal and vertical meridia, we selectively analyzed target locations *between* those used in the present study (i.e., upper left, upper right, lower left and lower right), taken from a previous unpublished data set from our laboratory. The data arise from an experiment employing similar methods to E2 (i.e., consecutive saccades to peripheral placeholders were signalled by central arrows), but that used 8 target locations surrounding fixation, and that did not involve a cue-back stimulus. In order to address concerns about the different lateralization of targets on the horizontal and vertical meridian, comparisons were made for 0° and 90° offset RTs for targets *within* only the left hemifield (i.e., comparing RTs to the upper-left vs. lower-left target) or right hemifield (i.e., upper-right vs. lower-right). The same comparison was made when targets were located in *different* hemifields (i.e., upper-left vs. upper-right, and lower-left vs. lower-right). The difference in RTs between 0° and 90° offset conditions was very similar, regardless of whether the S1 and S2 targets were located in the same hemifield (magnitude of IOR = 10.1 ms) or different hemifields (magnitude of IOR = 10.8 ms), F(1,19) = 0.27, *p* = .87.

One additional concern emerging from E2 is that the cue-back stimulus (which is aligned 180° opposite S1) could have somehow generated IOR for S2 responses also offset by 180° (e.g. through consecutive stimulation of the retinotopic location encoding both the cue-back and the 180° target location). We think this is unlikely for three reasons. First, the cue-back stimulus, which was present in both E1 and E2, did not produce IOR at 180° offsets in E1. Second, a previous unpublished study conducted in our lab was identical to E2, except that 8 target locations were possible and no cue-back stimulus was used. In that experiment, the same non-monotonic pattern of RTs was observed, despite the fact that no cue-back stimulus was used. Third, as will be seen, in E4 participants made consecutive arm movements to central stimuli while keeping their eyes at fixation; as a result, the retinotopic position of the cue-back stimulus would not overlap with targets offset by 180. However, both E2 and E4 reveal a similar non-monotonic pattern of result.

The next two experiments were conducted in order to extend the results of E1 and E2 to a different effector system. If signal type plays an important role in shaping the pattern of RTs observed, then the pattern of RTs observed should vary as a function of signal type, but be relatively independent of the effector system used to respond.

## Experiment 3: Peripheral Target–Arm

In E3, we required consecutive reaching movements to peripheral stimuli. If the use of peripheral stimuli is associated with the monotonic pattern of IOR, then a similar topography of RTs should be observed for E3 as compared to E1 (0°>90°>/ = 180°).

### Methods

#### Participants

Sixteen (12 female, 4 male) undergraduate students participated in E2. All participants were recruited through the Department of Psychology subject pool at Dalhousie University. All participants were right handed, had normal or corrected-to-normal vision and reported no history of visual, motor, or neurological abnormalities.

#### Apparatus, stimuli, procedure and data analysis

The apparatus, stimuli, procedure and data analyses were equivalent between E1 and E3 except that consecutive arm movements were required instead of eye movements. Participants began each trial by placing their right index finger at central fixation; responses to S1, the cue-back, and S2 were the same as in E1 and E2 except that participants were required to localize each target (and the fixation circle for the cue-back movements) by moving their arm to touch each marked location with their finger. Throughout each trial, participants were required keep their eyes at fixation. Reaction times were collected on the same 30-inch ELO touch screen LCD monitor (Elo TouchSystems, Menlo Park, California, USA) used in E1 and E2, and were defined by the moment participants lifted their finger from central fixation (relative to stimulus onset). If participants failed to keep their eyes at fixation, an error message was displayed, the trial was aborted, and not recycled. Similar to E1 and E2, if participants did not respond within 1.5 seconds to S1 or S2, if they did not return their finger to center between S1 and S2, or if they failed to keep their finger at center during the fixation intervals (immediately prior to S1 or S2), an error message was displayed, the trial was aborted, and was not recycled. Data from aborted trials were excluded from all subsequent analyses. Less than 5% of trials were aborted due to a slow response, a failure to return their finger to center between S1 and S2, or a failure to keep the eyes at fixation. The frequency of anticipation, miss, and directional errors (all removed from subsequent analyses) accounted for less than 5% of trials.

### Results

#### Errors

Directional errors accounted for 0.54%, 0.54%, and 0.71% of the total trials in the 0°, 90°, and 180° conditions respectively. Because error rates were less than 1% in each offset condition, they were not analyzed further.

#### Reaction time to S2

Reaching RTs are shown in Figure 2 (“Peripheral – Arm”). A main effect of offset was observed, *F*(2,30) = 25.24, p<0.001. Pairwise comparisons revealed slower RTs for 0° relative to 90°, *F*(1,15) = 58.14, p<0.001 and 180°, *F*(1,15) = 30.07, p<0.001. Reaction times were marginally slower for 90° compared to 180° offsets, *F*(1,15) = 4.34, p = .055.

Zero degree offset responses were significantly slower than 180° responses within both the horizontal, *F*(1,15) = 18.84, p<0.001 and vertical *F*(1,15) = 28.75, p<0.001 axes. No differences in the magnitude of IOR (where the magnitude of IOR = “same” S1/S2 RTs minus “different” S1/S2 RTs) was observed between any of the possible 90° offset combinations (UL, UR, LR, LL), *F*(3,45) = .38, p = 0.76, indicating that the overall faster RTs for the 90° offset condition were not driven by any specific 90° S1/S2 combination(s).

### Discussion

The results of E3 confirm that when consecutive arm movements are required to peripheral signals, the spatial topography of IOR is similar to the pattern of IOR observed in previous IOR tasks using peripheral stimuli [8]–[16]. Taken together, the results of E1 and E3 reveal for the first time that the monotonic pattern of RTs can be expected when a *target-target* task is used to prompt either consecutive eye or arm movements.

## Experiment 4: Central Target–Arm

In E4, we required consecutive reaching movements to peripheral placeholders as signalled by central stimuli. If the pattern of RTs observed in E2 (0° = 180° >90°) is related to the use of central stimuli, independent of the effector system used to respond, then a similar pattern of RTs should be observed in E4.

### Method

#### Participants

Twenty (13 female, 7 male) undergraduate students participated in E4. All participants were recruited through the Department of Psychology subject pool at Dalhousie University. Participants were right handed, had normal or corrected-to-normal vision and reported no history of visual, motor, or neurological abnormalities.

#### Apparatus, stimuli, procedure and data analysis

The apparatus, stimuli, procedure & data analyses were equivalent between E3 and E4 except that rather than using peripheral signals, arm movements were signalled using the same arrowheads used in E2 (Figure 1). The frequency of anticipation, miss, and directional errors (all removed from subsequent analyses) accounted for less than 5% of trials.

### Results

#### Errors

Directional errors accounted for 0.42%, 0.24%, and 0.35% of the total trials in the 0°, 90°, and 180° conditions respectively. Because error rates were less than 1% in each offset condition, they were not analyzed further.

#### Reaction time to S2

Reaching RTs are shown in Figure 2 (“Central – Arm”). A main effect of offset was observed, F(2,36) = 9.38, *p*<0.001. Pairwise comparisons revealed slower RTs for 0° relative to 90°, F(1,18) = 16.03, *p*<0.001 and 180° relative to 90°, *F*(1,18) = 10.17, *p*<0.005. RTs were not significantly different for 0° and 180° offsets, F(1,18) = 2.53, *p* = 0.13. In order to compare the spatial distribution of IOR observed in E4 to that observed in E3, we conducted a 2×3 mixed ANOVA with factors of signal type (peripheral or central) and offset (0°, 90°, and 180°). Significant main effects of signal type F(1,15) = 11.4, *p*<0.051, and offset, F(2,30) = 15.9, *p*<0.001, were observed. Moreover, a significant interaction between signal type and offset was observed F(2,30) = 6.7, *p*<0.005, indicating a difference in overall spatial topographies observed between experiments.

Within both the horizontal, F(1,18) = 2.44, *p* = .14, and vertical, F(1, 18) = .86, *p = *.37, axis, RTs did not differ for the 0° and 180° offset conditions. No differences in the magnitude of IOR (where the magnitude of IOR = “same” S1/S2 RTs minus “different” S1/S2 RTs) was observed between any of the possible 90° offset combinations (UL, UR, LR, LL; *F*(3,60) = 0.57, *p* = 0.63), indicating that the overall faster RTs for the 90° offset condition were not driven by a specific combination of first and second movement directions.

### Discussion

Like E2, significantly faster RTs were observed in the 90° offset condition relative to both the 0° and 180° offset conditions. Taken together, the results of E2 and E4 reveal that the non-monotonic pattern of RTs observed occurs independent of the effector system used to respond. Moreover, the non-monotonic pattern of RTs appears to depend on the use of central signals.

Previous work using central stimuli to examine manual responses to target locations aligned to the left and right of fixation have failed to observe IOR (e.g. [3], [17]). Consistent with those observations, we failed to observe a RT difference between the 0° and 180° offset conditions (in either the horizontal or vertical axis). Notably, E4 demonstrates that a response bias does in fact exist for consecutive manual localization responses made to central stimuli; however, previous studies might have missed this observation due to their use of only two target locations offset by 180°.

## General Discussion

Here we compared the pattern of RTs observed as a function of the angular offset between two consecutive eye or arm movement responses that were required to *either* peripheral or central signals. If IOR is present, the latency to initiate a saccade or reaching movement should be delayed by an amount of time that is related to its angular offset from a preceding movement [8]–[16]. In particular, based on the current spatial definition of IOR, RTs should decrease monotonically as the angular offset between the first and second stimuli increases from 0° to 180° (0°>90°>/ = 180°).

When peripheral stimuli were used to prompt either saccadic (E1) or reaching responses (E3), we replicated the monotonic pattern of RTs commonly observed in the IOR literature. In contrast, when we used central stimuli, we found a non-monotonic spatial topography of RTs for both saccades (E2) and reaching movements (E4), where responses were fastest for movements offset by 90° compared to either 0° or 180°.

### Defining IOR

The characteristics, possible mechanisms and functions, and indeed, the very definition of IOR is commonly debated with reference to the spatial distribution of RTs observed [8]–[16]. Notably however, this debate has occurred primarily in the context of experiments that used peripheral rather than central stimuli. The results of our experiments highlight the importance of examining the spatial topography of RTs, and provide an important reference point for future theories and studies of IOR. Indeed, the present results may be useful in attempts to clarify the definition of IOR, a phenomenon that is loosely ascribed to reaction time differences observed by scholars [25]. In this regard, an important question will regard whether or not the non-monotonic topography observed presently can be classified as IOR. On one hand, the non-monotonic topography violates existing characterizations of IOR as a behavioural phenomenon that selectively biases responses away from *previously signalled* locations, for example to facilitate visual search (e.g. [8], [10], [11]). On the other hand, it might be possible to argue that different forms of IOR can have different spatial distributions, (e.g. depending on signal type or other experimental manipulations), while being reconciled in other critical ways (e.g., if the different spatial distributions of RTs are implemented functionally, to prevent repetitive behaviours at different levels of sensorimotor processing). We anticipate that future research will be informative in this debate.

### Possible Mechanisms

What mechanisms might underlie the different spatial topographies of RTs observed? As mentioned in the general introduction, some scholars have suggested that IOR might affect response-based processes, (and in particular, motor based processes), when central rather than peripheral signals are used [3]–[7], [17]–[19]. The logic behind such claims rests on two interrelated observations. First, IOR is present when peripheral but *not* central targets follow a *peripheral* cue, and simple detection responses are required. This observation suggests that peripheral cues can generate a spatially restricted sensory processing deficit; notably, there is compelling neurophysiological evidence in support of this kind of IOR mechanism [23], [24]. Qualifying the first observation, IOR is present when *either* a peripheral or central target is used (regardless of cue type), *provided* a motor response is required to the target. (IOR appears to be present regardless of signal type, provided a motor response is made to the target, and regardless of whether or not a response is made to the cue, with one exception. When a central cue is used, IOR is observed for central and peripheral targets provided a motor response (either manual or saccadic) is made to *both* the cue and target [3]) Taken together, these two observations converge on the idea that primarily response-based processes are be affected by IOR (where present), when central signals are used.

If response-based processes (whether motor or late-stage attentional) are indeed isolated through the use of central stimuli, the range of possible mechanisms underlying the non-monotonic pattern of RTs in E2 and E4 is presumably limited to the neural mechanisms controlling the programming or execution of a response. Below we speculate a mechanism that can predict the pattern of results observed in E2 and E4.

In an fMRI study of arm movements instructed by central stimuli, we found evidence of directionally selective adaptation (i.e., reduction) of the BOLD response in several areas of human sensorimotor cortex, when consecutive movements were repeated in the same direction. Notably, adaptation only occurred for consecutive responses offset by 0° (i.e. when movements were made in the same direction), while a spatial offset of 90° or 180° between repeated movements did not reveal adaptation [26]. In the context of the center-out IOR task adopted presently, our fMRI results predict that neural adaptation will occur in the 0° and 180° spatial offset conditions, because both conditions require the repetition of a recently completed movement – either a repetition of the movement to the first target, or a repetition of the opposite, return-to-center movement. Given the relatively narrow tuning function for adaptation in most neurons (i.e., where movements offset by 90° show little to no adaptation) [26]–[28], our fMRI results predict that neural adaptation will be minimal for 90° offset conditions because the second target response is 90° away from both the first target movement and the return-to-center movement. Assuming that adaptation effects revealed by fMRI are associated with decreased neural firing rates and therefore processing efficiency (e.g., where it takes longer to reach response threshold), one would predict an increased response latency for conditions associated with the presence of adaptation. Indeed, the pattern of RTs observed in E2 and E4 is consistent with the neural adaptation mechanism described.

### The Relationship between Adaptation and Reaction Times

The neural adaptation model proposed is attractive because it can parsimoniously explain the spatial topography of RTs observed in E2 and E4. (Note that similar direction-encoding neurons are associated with the control of both reaching movements [26], [27], [29]–[31] and eye movements [32]–[37]; one would therefore expect similar adaptation effects in both effector systems.) Like any inference regarding the neurophysiological underpinnings of a behavioural phenomenon, the adaptation model relies on certain assumptions. The assumption that neurons encoding movement direction adapt following the execution of a single movement is well supported by fMRI research examining the control of movement direction in human motor cortex [26], [27]. This research has revealed the presence of directionally selective adaptation effects after the execution of a single movement. Indeed, the predominant tuning width of directional selectivity appears to be less than 90° [26]–[28]; therefore, adaptation effects likely only occur within neurons whose preferred directions are within 90° or less of the produced movement. As a consequence, S2 movements offset by 90° or more should engage neurons that were not adapted by the S1 or return-to-center movement. Key to the adaptation explanation of E2 and E4 is the additional assumption that a reduction of firing rates causes a delay in the time taken to reach response threshold and corresponding RTs. This idea is supported by research revealing that movement RTs can be predicted from neural firing rates [38], [39].

### Adaptation and Inhibition of Return for Consecutive Motor Responses

If adaptation effects are observed in participant RTs for consecutive movement responses, why are different spatial topographies of RTs observed when peripheral versus central stimuli are used, despite the fact that the movements required (to localize a peripheral placeholder) are the same in both cases? At least two options can explain this difference. First, it is possible that similar motor adaptation effects occur for both central and peripheral signals; however, in the case of peripheral stimuli, sensory/attentional effects (i.e., associated with detecting and processing spatial information about the target’s location) may also be present [23], [24]. Indeed, as demonstrated by Wang et al. [7], the use of peripheral stimuli in a target-target task (i.e. where participants respond to both the first and second signal, as in the present study) is likely to engage *both* sensory and motor-based effects that operate on different stages of processing. It is therefore likely that the spatial topography of RTs observed is determined by some combination of sensory/attentional and motor based effects when responses are made to peripheral stimuli. A second possibility is that responses to central and peripheral stimuli involve different populations of sensorimotor neurons and therefore result in independent adaptation effects. This is possible if adaptation occurs at earlier rather than later stages of sensorimotor processing, as the later stages of motor output are likely shared by responses regardless of the eliciting stimulus. Future studies that pair central-peripheral and peripheral-central targets might help to shed light on these possibilities.

### Spatial Attention and Motor Based Effects

As discussed, previous research has argued that motor-based effects in IOR may be isolated through the use of central rather than peripheral signals. This idea is well supported, however it is important to note that certain alternatives to this explanation may exist. In particular, if one assumes a tight link between the deployment of spatial attention and the planning or execution of an eye or arm movement, e.g., where spatial attention is deployed to the target of the movement immediately prior to execution, then it is possible that a late-stage attentional effect is involved in the response biases observed, *any* time a movement is planned [20], [21]. Given the use of a target-target task in the present study (where responses were required to both S1 and S2), it is therefore possible that the response biases observed are somehow associated with this late-stage attentional process. Notably however, because the deployment of late-stage attention is likely similar independent of signal type (and rather, dependent on the planning or execution of movement), if such an explanation is possible, it is not immediately clear how it could account for the different spatial topographies observed between E1/E3 and E2/E4.

### Reaction Times in the Vertical and Horizontal Axes

An interesting result emerging from our study is a difference in the comparison of 0° and 180° RTs observed in the horizontal axis, when eye versus arm movements were elicited by central stimuli (in E2 and E4 respectively). For eye movements, 180° offset responses were faster than 0° responses in the horizontal axis; this did not occur in the vertical axis (180° was similar to 0°), and it did not occur for arm movements in the horizontal axis (180° was similar to 0°). We suspect that the presence of a 180° RT advantage for consecutive eye movements in the horizontal axis might arise from learned behaviours such as scanning the horizon or reading [40]. If this is the case, then given that the arm is not necessarily specialized for movements in the horizontal axis, one would expect similar RT effects for consecutive arm movements in both the vertical and horizontal axis (as seen in E4). These observations highlight the importance of examining RTs across target locations aligned in different movement axes.

### Motor IOR in the Reaching System

The presence of motor IOR in the reaching control system remains controversial, and some authors have concluded that motor IOR is restricted to the oculomotor system (e.g., Fischer et al. [17]). In Fischer et al.’s study [17], only 0° and 180° target offsets were considered and reaction times for reaching movements were reported to be similar, which is also true for the 0° and 180° offsets in the present investigation. Of course, in the present investigation, 90° offsets were also included and RTs in this condition were found to be faster in comparison to both 0° and 180° offsets. Therefore, it is possible that Fischer et al. might also have observed some evidence of a spatially-tuned pattern of RTs in their experiment had additional spatial offsets been included. However, given that the pattern of RTs observed in the central-target conditions of our study do not resemble the current monotonic spatial definition of IOR, consistent with Fischer et al. [17], our study provides reason to question the idea that motor IOR can affect reaching responses to central stimuli.

### Conclusion & Future Directions

To our knowledge, the present study is the first to provide behavioural evidence of a non-monotonic pattern of RTs within a target-target IOR paradigm using central stimuli. An important question for future studies will be to determine whether the 90° RT advantage has any adaptive value, or if it is merely an epiphenomenon of neural adaptation (or perhaps some other mechanism involved in the control of movement). In any case, the fact that 90° offsets exhibit a performance advantage (characterized by faster RTs) when central stimuli are used, suggests that future studies of IOR should include more than the traditional 0° (same) and 180° (different) spatial offset conditions. Given that the monotonic pattern of RTs [8]–[16] has only been revealed in a select range of experimental conditions that might produce IOR [3], it would be useful for future research to establish the topography of RTs under these different experimental conditions. Indeed, an examination of the spatial distribution of RTs under these different task circumstances may be informative with respect to the ongoing debate surrounding the mechanism and function of IOR and other potentially related response biases. Finally, it is important for ongoing research to establish the different task circumstances that reveal different topographies of RTs. For example, it might be the case that the pattern of results observed is inherently related to the use of a center-out paradigm; future research interested in the mechanisms underlying orienting behaviour should use variants of the present paradigm to further address this and related questions.
